# Non-Stomatal Limitation to Photosynthesis in *Cinnamomum camphora* Seedings Exposed to Elevated O^3^

**DOI:** 10.1371/journal.pone.0098572

**Published:** 2014-06-03

**Authors:** Junfeng Niu, Zhaozhong Feng, Weiwei Zhang, Ping Zhao, Xiaoke Wang

**Affiliations:** 1 Key Laboratory of Vegetation Restoration and Management of Degraded Ecosystems, South China Botanical Garden, Chinese Academy of Sciences, Guangzhou, Guangdong, China; 2 State Key Laboratory of Urban and Regional Ecology, Research Center for Eco-Environmental Sciences, Chinese Academy of Sciences, Beijing, China; 3 Key Laboratory of Black Soil Ecology, Northeast Institute of Geography and Agroecology, Chinese Academy of Sciences, Harbin, Heilongjiang, China; Tennessee State University, United States of America

## Abstract

Ozone (O_3_) is the most phytotoxic air pollutant for global forests, with decreased photosynthesis widely regarded as one of its most common effects. However, controversy exists concerning the mechanism that underlies the depressing effects of O_3_ on CO_2_ assimilation. In the present study, seedlings of *Cinnamomum camphora*, a subtropical evergreen tree species that has rarely been studied, were exposed to ambient air (AA), ambient air plus 60 [ppb] O_3_ (AA+60), or ambient air plus 120 [ppb] O_3_ (AA+120) in open-top chambers (OTCs) for 2 years. Photosynthetic CO_2_ exchange and chlorophyll a fluorescence were investigated in the second growing season (2010). We aim to determine whether stomatal or non-stomatal limitation is responsible for the photosynthesis reduction and to explore the potential implications for forest ecosystem functions. Results indicate that elevated O_3_ (E-O_3_) reduced the net photosynthetic rates (*P*_N_) by 6.0-32.2%, with significant differences between AA+60 and AA+120 and across the four measurement campaigns (MCs). The actual photochemical efficiency of photosystem II (PSII) in saturated light (F_v_^′^/F_m_^′^) was also significantly decreased by E-O_3_, as was the effective quantum yield of PSII photochemistry (Φ_PSII_). Moreover, E-O_3_ significantly and negatively impacted the maximum rates of carboxylation (*V*_cmax_) and electron transport (*J*_max_). Although neither the stomatal conductance (*g*_s_) nor the intercellular CO_2_ concentration (*C*_i_) was decreased by E-O_3_, *P*_N_/*g*_s_ was significantly reduced. Therefore, the observed reduction in *P*_N_ in the present study should not be attributed to the unavailability of CO_2_ due to stomatal limitation, but rather to the O_3_-induced damage to Ribulose-1,5-bisphosphate carboxylase/oxygenase and the photochemical apparatus. This suggests that the down-regulation of stomatal conductance could fail to occur, and the biochemical processes in protoplasts would become more susceptible to injuries under long-term O_3_ exposure, which may have important consequences for forest carbon and water budget.

## Introduction

Of the known phytotoxic air pollutants, ozone (O_3_) has the greatest potential to detrimentally impact forests [1]. The negative effects of O_3_ on plants are commonly attributed to its highly oxidative properties that can damage cell membranes, denature critical enzymes, and give rise to various reactive oxygen species (ROS) [2], . One of the most consistent effects of O_3_ on trees is the inhibition of photosynthesis [5], [6], [7]. Based on a quantitative meta-analysis of over 100 studies of O_3_ effect on trees, it has been estimated that an 11% decrease in net photosynthetic rates (*P*_N_) has resulted from the increase in O_3_ levels that has occurred since the Industrial Revolution [8].

However, controversy exists concerning the mechanism that underlies the depressing effects of O_3_ on CO_2_ assimilation in plants [9]. While previous studies have frequently linked these effects to decreased stomatal conductance (*g*_s_), many others have related the O_3_-induced decline in photosynthesis to altered mesophyll activities, such as reduced maximum rates of carboxylation (*V*_cmax_) and electron transport (*J*_max_) [10], [11], [12], [13]. Additionally, under high levels of O_3_, changes in chlorophyll a fluorescence, including reductions in photochemical quenching (qP), actual photochemical efficiency (F_v_^′^/F_m_^′^) and the effective quantum yield of photosystem II (PSII) in saturated light (Ф_PSII_), have also been widely reported [14],[15]. In fact, due to its high-resolution, real-time, and non-invasive nature, chlorophyll a fluorescence measurement has become an important technique parallel to gas exchange analyses for confirming the primary site of photosynthetic limitation [16]. Combined measurements of chlorophyll a fluorescence and gas exchange can provide valuable information regarding plant photosynthetic performance [17].

Field and experimental evidence suggests that broadleaf evergreen trees in Mediterranean areas are comparatively more O_3_ tolerant than deciduous species in temperate and boreal regions [18], [19]. This can be largely attributed to the sclerophyllous traits of the former, as well as the uniquely Mediterranean climate that concentrates high levels of O_3_ during seasons of drought, which triggers stomatal closure [20], [21], [22], [23]. In subtropical regions, trees also develop glossy and leathery leaves, however, the climate is generally characterized by four clearly demarcated seasons with rain and heat co-occurring in summers, and so far very little information is available concerning the impact of O_3_ on the regional evergreen tree species.

In the present study, seedlings of *C. camphora*, a subtropical evergreen broadleaf tree species native to the Yangtze River Delta in eastern China, were exposed to ambient air (AA), ambient air plus 60 [ppb] O_3_ (AA+60), or ambient air plus 120 [ppb] O_3_ (AA+120) in open-top chambers (OTCs) for 2 years. During the second growing season (2010), gas exchange and chlorophyll a fluorescence were measured and analyzed. The aims of this experiment were to: (1) determine the extent to which photosynthesis is reduced in the experimental seedlings exposed to elevated O_3_ (E-O_3_, AA+60 or AA+120), (2) clarify whether stomatal or non-stomatal limitation is responsible for this reduction, and (3) explore the ecological meaning of our findings to broad-scale studies.

## Materials and Methods

### Experimental site and plant material

Permits and approvals for the work were obtained from East China Normal University and Tiantong Forest Farm, which are responsible for the protection of the Tiantong National Forest Park. The experiment was carried out within the park, at the Tiantong National Field Observation and Research Station for Forest Ecosystems (29°48′N, 121°47′E), Ningbo, Zhejiang province, China. One-year-old *C. camphora* seedlings, a typical subtropical evergreen broadleaf tree species widely distributed throughout eastern China and Japan, were planted in 5-[L] plastic pots and fumigated with E-O_3_ for 2 years. These seedlings were purchased from a nearby commercial nursery and selected for phenotypic homogeneity. The potting soil consisted of a mixture of native yellowish-brown lateritic soil and litter collected from a fir forest at a 1∶1 ratio. All seedlings were acclimated to OTC conditions for two weeks before O_3_ fumigation. More information about the climate conditions of the experimental site and the plant cultivation prior to O_3_ treatment were described in Niu *et al*.[24].

### OTCs and treatments

Six OTCs (octagonal base, 5.5 [m^2^] of basal area, and 2.6 [m] in height) were set up at the experimental field in early 2009. The rate of light transmittance of the OTCs was 98.3% and the average air velocity corresponded to a turnover rate of two complete air changes per minute. O_3_ was generated from pure oxygen using an electrical discharge O_3_ generator (*HY003*, *Chuangcheng Technology Co., Ltd*., Jinan, China) and piped into four of the six OTCs in mixture with ambient air. O_3_ flow was modulated using mass flow meters (*SY-9311, Beijing Shengye Science and Technology Development Co., Ltd.*, Beijing, China) in order to obtain the designated O_3_ concentration within each OTC. The seedlings were fumigated from 9:00–17:00, 7 days per week, 25 May to 10 September 2009 and 1 May to 7 October 2010, except for rainy and mostly cloudy days.

Replicate AA, AA+60, and AA+120 chambers were randomly arranged in the experimental field. Seedling positions within each OTC were changed every 3-5 days. Every 10-15 days, all chambers were emptied and randomly reassigned a new O_3_ level, and the seedlings were replaced according to their specified treatment levels. This allowed us to eliminate position and chamber effects, treating each plant as an independent experimental unit. Five seedlings within each OTC and a total of 30 plants (5 plants×3 groups×2 OTCs) were investigated.

### Measurements

Temperature and relative air humidity were recorded at 30-minute intervals using thermo-hygrographs (*DSR-TH, ZOGLAB Microsystem Inc.*, Hangzhou, China) inside and outside the OTCs. O_3_ concentrations at approximately 10 [cm] above the plant canopy were monitored using a UV-absorption O_3_ analyzer (*Model 49i, Thermo Scientific Inc*., Connecticut, USA).

Gas exchange and chlorophyll a fluorescence under light conditions were measured using an infrared gas analyzer (IRGA) fitted with a 6400-40 leaf chamber fluorometer (*LI-6400, LI-COR Inc.*, Lincoln, NE, USA). All measurements were made from 9:00–12:00 and recorded when the coefficient of variance (CV) was less than 3%. The photosynthetic photon flux density (PPFD) was fixed at a saturating intensity of 1200 [µmol m^−2^ s^−1^]. CO_2_ was supplied with pure CO_2_ cylinders and maintained at 380 [µmol mol^−1^]. Block temperature of the cuvette was set to 30±0.5°C, and relative humidity 60±5%. Maximum, minimum and steady state fluorescence under light conditions (F_m_^′^, F_o_^′^ and F_s_) were measured, and Ф_PSII_, qP and F_v_^′^/F_m_^′^ were calculated as (F_m_^′^-F_s_)/F_m_^′^, (F_m_^′^-F_s_)/(F_m_^′^-F_o_^′^) and (F_m_^′^- F_o_^′^)/F_m_^′^, respectively. Six plants per treatment were analyzed, and only fully expanded upper leaves were screened. Tracking analyses of leaves in the same leaf position were carried out monthly (2 July, 7 August, 7 September, and 8 October 2010).

In order to determine the maximum photochemical efficiency of PSII (F_v_/F_m_), a field-portable chlorophyll fluorometer (*FMS 2*, *Hansatech Instruments Ltd*., Norfolk, UK) was employed. The same leaves used for gas exchange analyses were screened. Leaves were adapted to dark for 30 minutes and the minimum fluorescence (F_o_) was measured by switching on the modulating light (0.6 [kHz]). Then, the application of a saturating light pulse (8000 [m^−2^ s^−1^] for 1 [s]) led to the rapid closure of PSII reaction centers, yielding the maximum fluorescence (F_m_). F_v_/F_m_ was calculated as (F_m_-F_o_)/F_m_.

The response of carbon assimilation rates to changing CO_2_ concentrations (*A*-*C*_i_ response curves) was determined by sequentially measuring the rates of photosynthesis at CO_2_ concentrations of 380, 200, 150, 100, 50, 400, 600, 900, 1200 and 1500 [µmol mol^−1^]. Light intensity, block temperature and relative humidity were set equal to those used in gas exchange analyses. *V*_cmax_ and *J*_max_ were determined and adjusted to 25°C according to Long and Bernacchi [25]. Four seedlings per treatment were screened, and two measurement campaigns (MCs) were carried out for the *A*-*C*_i_ response curves, on 22 July and 24 September 2010, respectively.

### Data analysis

Data were analyzed using SAS software (*Version 9.1.3*, *SAS Institute*, Cary, NC, USA). Repeated measures analyses of variance (RANOVAs) were performed in order to analyze the overall effect of O_3_ on the examined parameters throughout the growing season. Variances across MCs, as well as the interaction between O_3_ and MCs were also investigated. For each MC, ANOVA model was applied to test the O_3_ effect and Bonferroni methods were adopted for post-hoc multiple comparisons. Normality of distribution and homogeneity of variance were tested before all analyses. Differences between treatments were considered significant if *p*≤0.05.

## Results

### OTC microclimate and O_3_ monitoring

Table 1 shows AOT40s (accumulated O_3_ exposure over a threshold of 40 [ppb]) and SUM60s (sum of hourly O_3_ concentration when the concentration is equal to or greater than 60 [ppb]) during each MC of gas exchange and chlorophyll a fluorescence during the 2010 growing season. Because of persistent rain (19 days) from 7 August to 7 September, the accumulation of AOT40 and SUM60 was lower during this period, as shown in Table 1. Under AA, the total dose of O_3_ was 6.7 and 8.7 [ppm h] in the forms of AOT40 and SUM60, respectively. AOT40s were generally lower than values expressed as SUM60s in all O_3_ regimes. Detailed descriptions of the average O_3_ concentrations, as well as the OTC microclimate conditions throughout the 2 years of this experiment can be found elsewhere [24], [26].

**Table 1:** AOT40s and SUM60s under different O_3_ exposure regimes during the 2010 growing season of *Cinnamomum camphora* seedlings.

	AOT40 [ppm h]^b^				SUM60 [ppm h]^c^			
O_3_ regimes^a^	2 July	7 Aug.	7 Sept.	8 Oct.	2 July	7 Aug.	7 Sept.	8 Oct.
AA	3.3	4.8	5.5	6.7	3.9	5.7	6.7	8.7
AA+60	12.9	19.1	21.7	26.1	14.3	26.6	32.3	45.6
AA+120	23.1	42.8	47.3	56.3	35.4	48.2	56.9	74.9

a AA: ambient air; AA+60: ambient air plus 60 [ppb] O_3_; AA+120: ambient air plus 120 [ppb] O_3_.

b AOT40 [ppm h]: accumulated O_3_ exposure over a threshold of 40 [ppb].

c SUM60 [ppm h]: sum of hourly O_3_ concentration when the concentration is equal to or greater than 60 [ppb].

### Gas exchange

**Figure 1:**
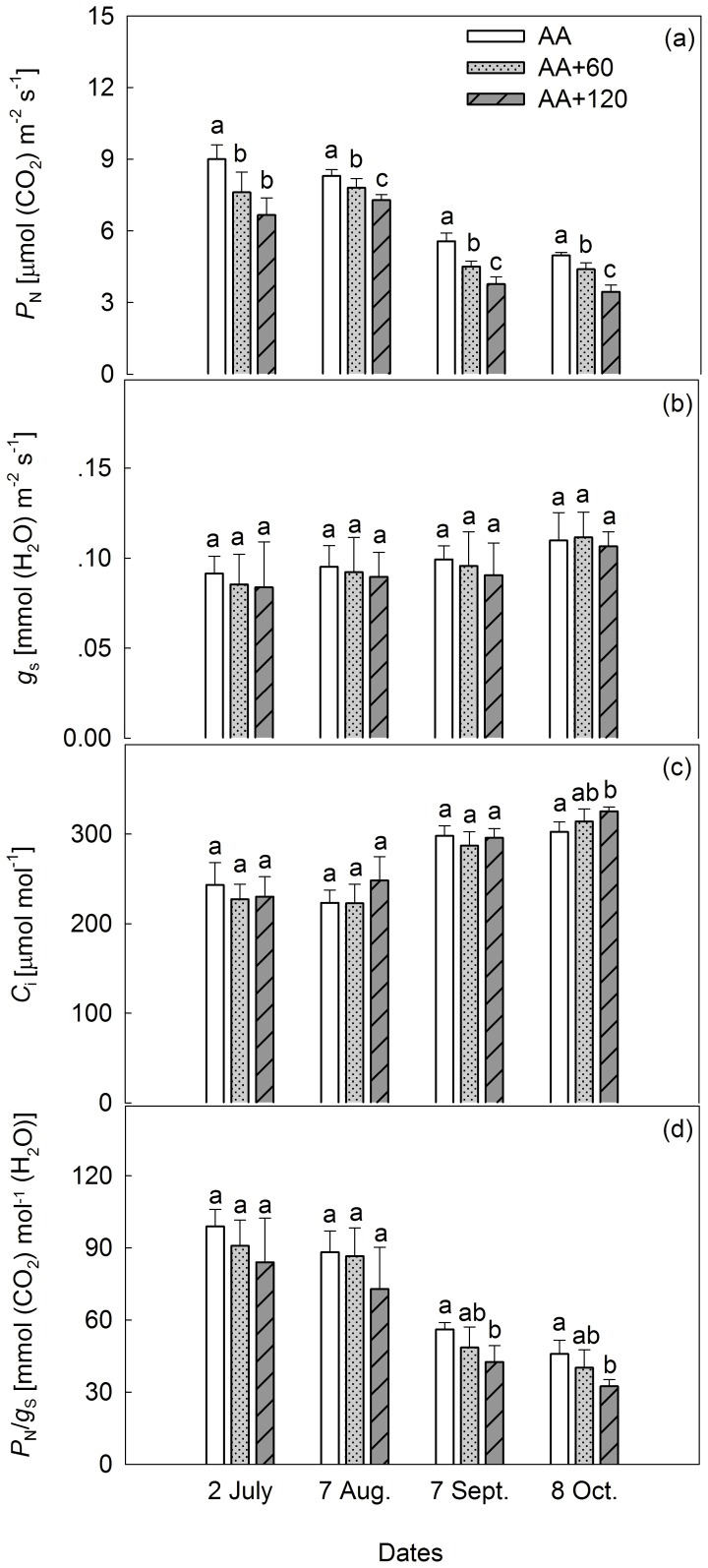
Effects of elevated O_3_ on gas exchange parameters. Vertical bars represent average levels and distinct letters indicate significant differences among O_3_ regimes (*n* = 6) (AA: ambient air; AA+60: ambient air plus 60 [ppb] O_3_; AA+120: ambient air plus 120 [ppb] O_3_).

Throughout the 2010 growing season, *P*_N_ was reduced, on average, by 13.0% and 25.3% under AA+60 and AA+120, respectively. Differences between these two treatment regimes were statistically significant, except on 2 July (Table 2 and Figure 1a). *P*_N_/*g*_s_ was also significantly reduced, while *C*_i_ was significantly increased by AA+120 on 8 October. A negative O_3_ effect on *P*_N_/*g*_s_ was also observed on 7 September (Figure 1d). Variations of *P*_N_, *g*_s_, *C*_i,_ and *P*_N_/*g*_s_ across the four MCs were statistically significant, and O_3_ interacted significantly with MCs for *P*_N_ and *C*_i_ (Table 2).

**Table 2:** Repeated measures ANOVAs (RANOVAs) of the gas exchange and chlorophyll a fluorescence parameters of *Cinnamomum camphora* seedlings during the 2010 growing season (*P* values are shown, *n* = 4 for *V*_cmax_, *J*_max_ and *J*_max_/*V*_cmax_, n = 6 for other parameters).

	Parameters	O_3_	MCs	O_3_×MCs^a^
Gas exchange	*P*_N_ [µmol (CO_2_) m^−2^ s^−1^]	**<.0001**	**<.0001**	**0.0169**
	*g*_s_ [mmol (H_2_O) m^−2^ s^−1^]	0.5738	**0.0001**	0.9956
	*C*_i_ [µmol mol^−1^]	0.1755	**<.0001**	**0.0377**
	*P*_N_/*g*_s_ [mmol (CO_2_) mol^−1^ (H_2_O)]	**0.0072**	**<.0001**	0.9435
	*V*_cmax_ [µmol m^−2^ s^−1^]	**0.0031**	0.3435	0.1481
	*J*_max_ [µmol m^−2^ s^−1^]	**<.0001**	**0.0007**	**0.0005**
	*J*_max_/*V*_cmax_	0.4994	0.1172	0.3419
Chlorophyll a fluorescence	F_v_^′^/F_m_^′^	**0.0310**	**0.0011**	0.1329
	Ф_PSII_	**<.0001**	**<.0001**	0.6421
	qP	**<.0001**	**<.0001**	0.3827
	F_v_/F_m_	0.5042	**<.0001**	0.9998

a MCs: measurement campaigns.

**Figure 2:**
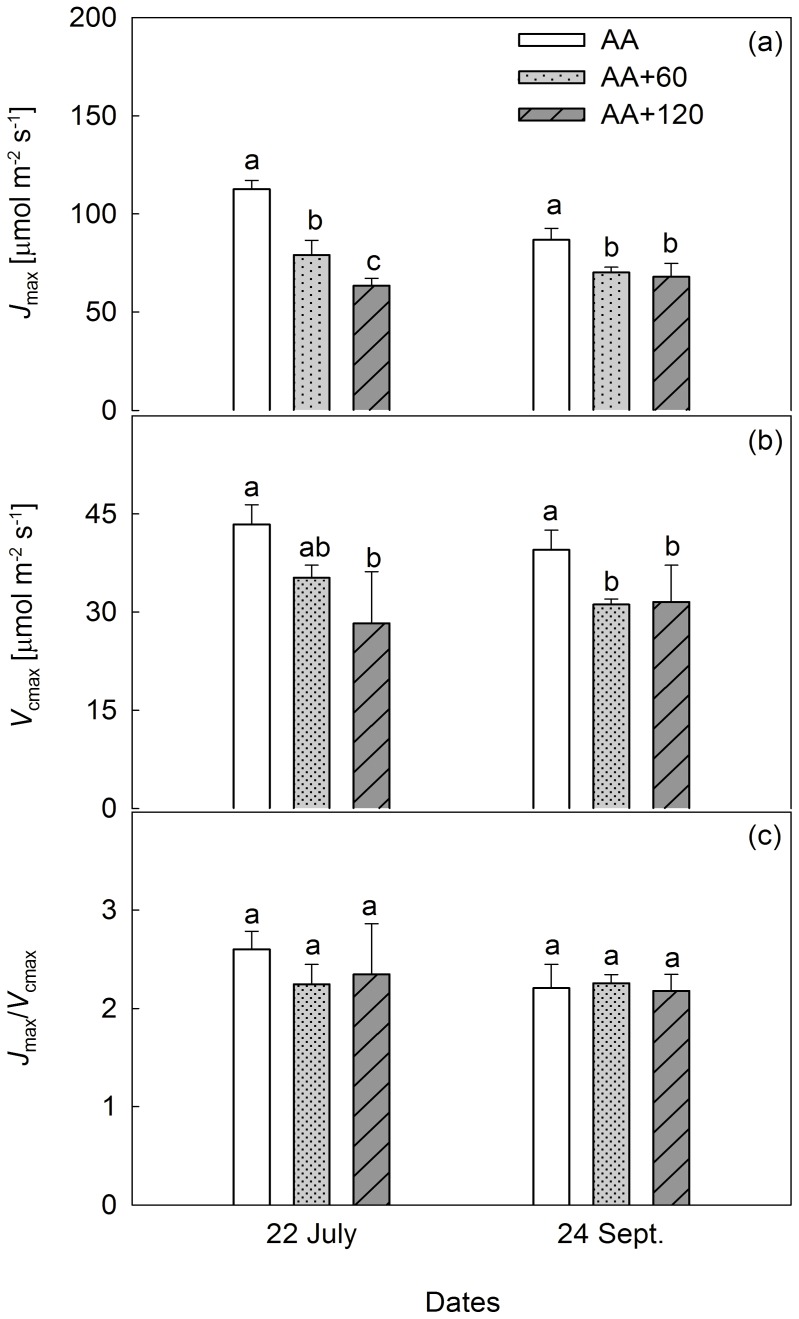
Effects of elevated O_3_ on the maximum rates of carboxylation (*V*_cmax_) and electron transport (*J*_max_). Vertical bars represent average levels and distinct letters indicate significant differences among O_3_ regimes (*n* = 4) (AA: ambient air; AA+60: ambient air plus 60 [ppb] O_3_; AA+120: ambient air plus 120 [ppb] O_3_).

*V*_cmax_ was significantly decreased by AA+60 and AA+120 on 24 September, but only by AA+120 on 22 July (Figure 2b). Both AA+60 and AA+120 exerted significantly negative effects on *J*_max_ (Figure 2a) across the two MCs. The difference in *J*_max_ between AA+60 and AA+120 was statistically significant on 22 July, but not on 24 September (Figure 2a). *J*_max_ varied significantly, while *V*_cmax_ maintained the same levels across the two MCs (Table 2). *J*_max_/*V*_cmax_ was not significantly affected by E-O_3_ in the present study (Figure 2c).

### Chlorophyll a fluorescence

**Figure 3:**
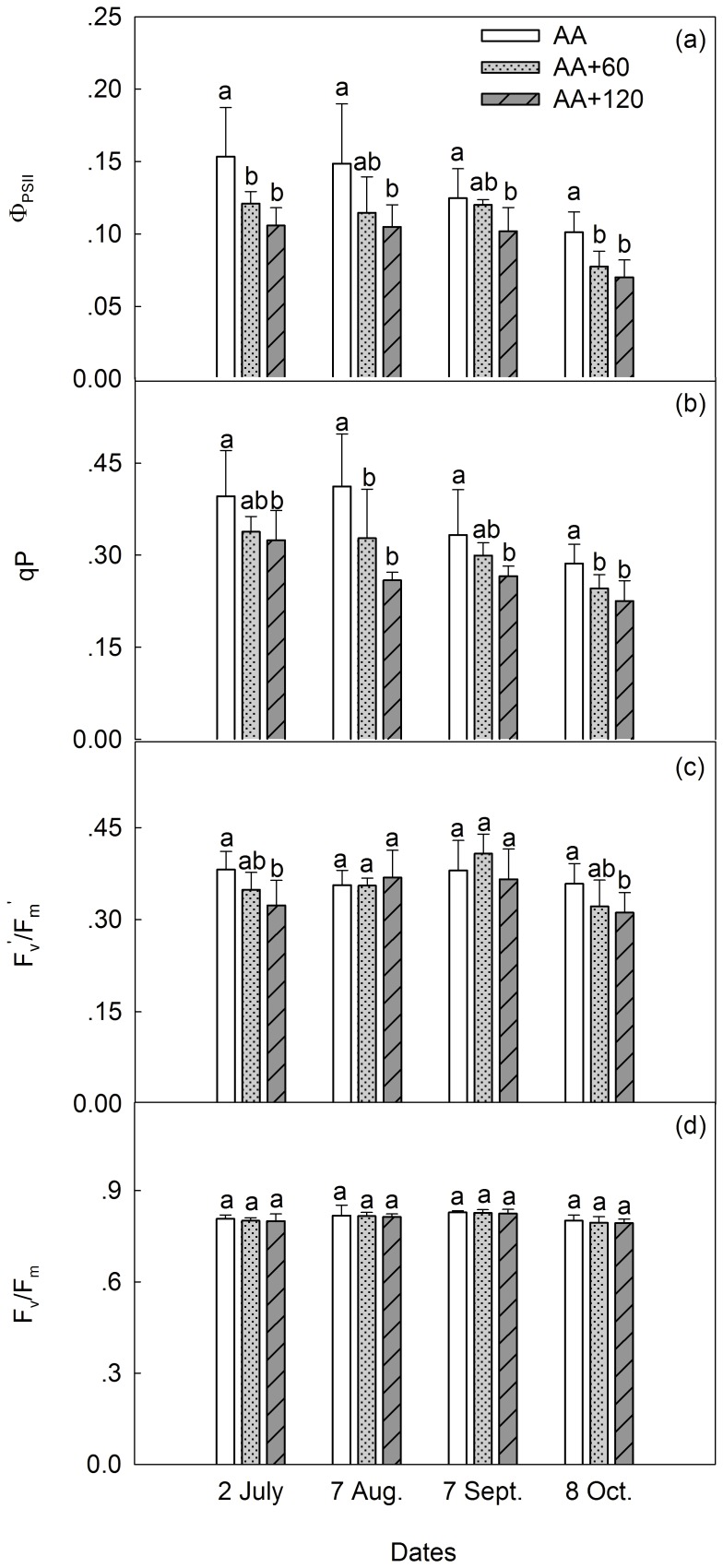
Effects of elevated O_3_ on the chlorophyll a fluorescence parameters. Vertical bars represent average levels and distinct letters indicate significant differences among O_3_ regimes (*n* = 6) (AA: ambient air; AA+60: ambient air plus 60 [ppb] O_3_; AA+120: ambient air plus 120 [ppb] O_3_).

F_v_^′^/F_m_^′^ and Ф_PSII_ were significantly decreased by E-O_3_ (Table 2). Ф_PSII_ was significantly decreased by AA+120 across all four MCs, and also by AA+60 on 2 July and 8 October. For F_v_^′^/F_m_^′^, only AA+120 exerted significant impact, on 2 July and 8 October (Figure 3a, c). Additionally, qP was significantly depressed by AA+120 across all four MCs, and also by AA+60 on 7 August and 8 October (Figure 3b). However, F_v_/F_m_ was not significantly influenced by E-O_3_ (Figure 3d). Differences across MCs were statistically significant for all fluorescence parameters. However, no interactions were found between O_3_ and MCs (Table 2).

## Discussion

E-O_3_ significantly reduced *P*_N_ in *C. camphora* over the course of the present study. At the end of the 2010 growing season, AA+60, which corresponded to an AOT40 of 26.1 [ppm h], reduced *P*_N_ by 11.7%. A similar 11.4% decrease in *P*_N_ occurred with an AOT40 of 36.2 [ppm h] in the Mediterranean evergreen Satsuma mandarin (*Cirtus unshiu* [Mak.] Marc.) [15]. However, in deciduous *Quercus pyrenaica*, *Quercus robur* and *Quercus faginea*, an AOT40 of 26.2-28.8 [ppm h] decreased *P*_N_ by 64%, 38% and 33%, respectively [27]. Based on the comparisons with these results, our findings suggest that *C. camphora* is less resistant to O_3_ than Mediterranean evergreen broadleaves, but more tolerant than deciduous species [7], [19]. Reduction in *P*_N_ under E-O_3_ has also been reported in other species, deciduous (*Quercus serrata*, *Populus tremuloides* Michx., *Betula pendula* Roth.) as well as evergreen (*Pinus taeda* L.) [28], [29], [30], [31], and therefore this may represent a common response behavior to high levels of O_3_ in woody plants [32].

Similar to observations in European beech (*Fagus sylvatica*) and black aspen (*Populus nigra*) [33], *g*_s_ was not affected by E-O_3_ in the present study (Figure 1b), indicating that the significant reduction in *P*_N_ of *C. camphora* cannot be attributed to stomatal behavior. Additionally, *C*_i_ was not reduced, but in fact significantly increased by AA+120 on 8 October, confirming that CO_2_ supply was not the limiting factor in reducing *P*_N_. Moreover, significant decrease in *P*_N_/*g*_s_ was detected under AA+120 on 7 September and 8 October, further suggesting that factors other than *g*_s_ should be considered when attempting to clarify the mechanism responsible for the reduction in *P*_N_ that results from O_3_ stress. Increased *C*_i_, as well as the negative relationship between *P*_N_ and *C*_i_ under elevated O_3_, has also been documented in previous literature [34], [35].

RuBisCO is commonly regarded as one of the primary targets of O_3_-induced damage [36], [37]. On both 22 July and 24 September, AA+120 significantly reduced *V*_cmax_, which was also notably decreased by AA+60 on 24 September (Figure 2b). These findings concur broadly with those reported in aspen (*P. tremuloides*) and birch (*B. pendula* Roth.) [28], [38], [39]. Decreases in RuBisCO quantity and activity may be responsible for the decline of *V*_cmax_ under E-O_3_ [40]. *J*_max_ was also significantly decreased by E-O_3_ in the present study, while *J*_max_/*V*_cmax_ was not affected (Figure 2c). The constant *J*_max_/*V*_cmax_ ratio indicates the close coupling between RuBP carboxylation and light-driven electron transport. Activities of these two processes are commonly related, and vary in parallel with environmental conditions [41]; thus the observed decrease in *J*_max_ might have occurred in response to the declining *V*_cmax_ [42]. Therefore, O_3_-induced degradation and deactivation of RuBisCO, as well as its feedback inhibitory effect on the electron transport system, might be a primary cause of the reduction in *P*_N_.

Confirming the previous findings in evergreen Mediterranean species [43], [44], F_v_/F_m_ in *C. camphora* was not influenced by E-O_3_ (Figure 3d). However, Fv′/Fm′ was significantly reduced by AA+120 on 2 July and 8 October, implying enhanced energy decay via non-radiative processes at the PSII reaction centers [45]. Meanwhile, in the present study, significant reductions of qP (notably under AA+120 at all four MCs and under AA+60 on 7 August and 8 October) and Ф_PSII_ (notably under AA+120 at all four MCs and under AA+60 on 2 July and 8 October) were also observed under E-O_3_ (Figure 3a, b). Similar results have been reported in Scots pine (*Pinus sylvestris* L.) and Satsuma mandarin (*C. unshiu* [Mak.] Marc.) [15], [17]. Reduction in qP and Ф_PSII_ may correlate with a decrease in the proportion of available excitation energy used in the photochemistry [11]. Decreased photochemistry but enhanced non-radiative decay suggests that more energy absorbed by PSII is dissipated as heat, and this could then lead to the overheating of PSII reaction centers. Therefore, direct oxidative damage, as well as indirect heat-related injuries to the photochemical apparatus, may also play an important role in mediating the down-regulation of *P*_N_ in *C. camphora* exposed to E-O_3_.

Previous studies have frequently linked the O_3_-induced decline in plant photosynthesis to its inhibitory effect on foliar stomatal conductance. Torsethaugen *et al.* found that acute O_3_ exposure inhibited the guard cell K^+^ channels, which mediate K^+^ uptake that drives stomatal opening, and thus led to decreased photosynthesis in *Vicia faba* [46]. Zhang *et al*. reported that the reduction in photosynthesis (-27%) of *Liriodendron chinense* (Hemsl.) Sarg seedlings was accompanied by a significant decrease of stomatal conductance (-34.7%) after O_3_ exposure for 40 days at a concentration of [150 ppb] [47]. During the first growing season (2009) of the present study, we also observed concurrent reduction in photosynthesis (-24.6%) and stomatal conductance (-34.2%) under [AA+60] [26], which was however not observed during the second growing season (2010). This suggests that the coupling between photosynthesis and stomatal conductance in plants could fail and the biochemical processes in protoplasts would become more susceptible to injuries under long-term O_3_ exposure.

Decoupling between photosynthesis and stomatal conductance under elevated O_3_ may have important implications for water use and carbon cycling of forest ecosystems [48]. One the one hand, failure or sluggishness of stomatal closure could give rise to excessive plant transpiration [49], resulting in unnecessary water loss, leading to regional water shortage, or even causing tree wilt and dieback if soil water supply is particularly tight, especially in arid and semi-arid areas. At the same time, increased exposure of mesophyll cells to O_3_ through open stoma, on the other hand, could decrease the efficiency of light use by photosystem II for CO_2_ assimilation [25], resulting in lower forest carbon sequestration and leading to a warmer atmosphere. Therefore, the potential impact of O_3_ under both current and future enriched conditions should be considered adequately in carbon budget calculations, forest hydrology simulations and climate change predictions at regional and global scales.

It should be noted that the present study was conducted on just one subtropical evergreen species of 2 to 3 years age. The unique physiological characteristics of seedlings and the optimal water status under OTC conditions as well as the restriction of root growth in pots may bias tree performance [4], [24]. To attain a comprehensive understanding of the effect of O_3_ on forests, as well as forest responses and feedbacks to global changes, further investigations based on mature trees of a wider range of other species are critically needed.

## Conclusions

E-O_3_ (AA+60 or AA+120) significantly reduced *P*_N_ in *C. camphora*. Comparisons of this reduction with those observed in other species suggest that *C. camphora* is less tolerant to O_3_ than Mediterranean evergreen trees, but more resistant than deciduous species. Reduction of stomatal conductance is not a reasonable explanation for the decline of *P*_N_ in the present study, as manifested by the increased *C*_i_ and decreased *P*_N_/*g*_s_. As with *P*_N_, decreases in *V*_cmax_, *J*_max_, Ф_PSII_ and qP were detected, indicating that direct oxidative damage and indirect heat-related injuries to RuBisCO and photochemical apparatus were responsible for the reduction in *P*_N_ that was observed in *C. camphora* under E-O_3_. This suggests that the biochemical processes in protoplasts will become more susceptible to injuries under long-term O_3_ exposure, which may bear important implications for forest water use and carbon cycling.
